# Fault Diagnosis Based on Chemical Sensor Data with an Active Deep Neural Network

**DOI:** 10.3390/s16101695

**Published:** 2016-10-13

**Authors:** Peng Jiang, Zhixin Hu, Jun Liu, Shanen Yu, Feng Wu

**Affiliations:** 1 College of Automation, Hangzhou Dianzi University, 310018 Hangzhou, China; hduhzx@163.com (Z.H.); shanen_yu@hdu.edu.cn (S.Y.); fengwu@hdu.edu.cn (F.W.); 2 State Key Laboratory of Industrial Control Technology, Institute of Industrial Process Control, Zhejiang University, 310027 Hangzhou, China; liujun@163.com

**Keywords:** fault diagnosis, deep learning, deep neural network, active learning, big sensor data

## Abstract

Big sensor data provide significant potential for chemical fault diagnosis, which involves the baseline values of security, stability and reliability in chemical processes. A deep neural network (DNN) with novel active learning for inducing chemical fault diagnosis is presented in this study. It is a method using large amount of chemical sensor data, which is a combination of deep learning and active learning criterion to target the difficulty of consecutive fault diagnosis. DNN with deep architectures, instead of shallow ones, could be developed through deep learning to learn a suitable feature representation from raw sensor data in an unsupervised manner using stacked denoising auto-encoder (SDAE) and work through a layer-by-layer successive learning process. The features are added to the top Softmax regression layer to construct the discriminative fault characteristics for diagnosis in a supervised manner. Considering the expensive and time consuming labeling of sensor data in chemical applications, in contrast to the available methods, we employ a novel active learning criterion for the particularity of chemical processes, which is a combination of Best vs. Second Best criterion (BvSB) and a Lowest False Positive criterion (LFP), for further fine-tuning of diagnosis model in an active manner rather than passive manner. That is, we allow models to rank the most informative sensor data to be labeled for updating the DNN parameters during the interaction phase. The effectiveness of the proposed method is validated in two well-known industrial datasets. Results indicate that the proposed method can obtain superior diagnosis accuracy and provide significant performance improvement in accuracy and false positive rate with less labeled chemical sensor data by further active learning compared with existing methods.

## 1. Introduction

Chemical industries have always been concerned about methods for reducing the risk of accidents because they may commonly occur in extreme environments, such as extraordinarily high temperature or pressure, which may result in public damage and large economic losses [1]. Accordingly, the chemical industry is highly supervised, where effective fault diagnosis provides the baseline values of security, stability and reliability, since fault diagnosis has been addressed as one of the best methods to prevent industry accidents [2,3]. Modern chemical processes have become more complex with the development of science and technology, and large amounts of data are being produced. The data can be analyzed to learn whether a fault has occurred in chemical processes, while determining significant potential in chemical fault diagnosis. The advancement of sensor technology has lessened the difficulties in the acquisition of data [4], namely, chemical sensor data, which implies the necessity of an effective fault diagnosis method to monitor the entire process and detect the fault in time by mining potential information from the large amounts of sensor data collected. 

Three types of methods are currently used in fault diagnosis from data processing perspective, namely model-based, signal-based and knowledge-based methods [5]. Model-based methods estimate the output of the system by constructing a model and achieving fault diagnosis through the residual between estimates and measurements. Methods of this type, such as parameter estimation and parity space methods, provide in-depth analysis for the dynamic of systems [6,7]. Given the complexity of modern chemical systems, explicitly representing the real chemical process with a precise mathematical model is complicated. Signal-based methods are based on the analysis of output signals, which address the problem of complex modeling. Typical signal analysis techniques, including fast Fourier transform, spectral estimation and wavelet transform, are employed in this type of methods [5]. Signal-based methods require thorough analysis and a priori knowledge on fault mechanism. In addition, the manually extracted feature has a limitation in terms of application, that is, it is only suitable for specific diagnosis issues, thus limiting the application in complex chemical systems [8].

As a principal part of artificial intelligence techniques, machine learning techniques, which are the main part of knowledge-based methods, have shown significant potential in fault diagnosis. This method is also called intelligent fault diagnosis method, where artificial intelligence techniques are combined [9]. This method attempts to acquire underlying knowledge from large amounts of empirical data through model learning and is more desirable than other methods [8]. As representatives, artificial neural network (ANN), support vector machine (SVM), and multi-layer perceptron (MLP) have been applied successfully in the field of fault diagnosis [10,11,12,13]. This type of method commonly combines signal processing techniques for initial feature extraction from signals. Amar et al. [14] extracted an image feature of the vibration spectrum and employed ANN to detect faults. Bin et al. [15] presented a method that provides wavelet packets-empirical mode decomposition for characteristics extraction and MLP network for fault classification. Luis et al. [16] achieved fault detection in the petroleum industry using one-class SVM. The representative features from signal processing and adaptive learning capability of machine learning algorithm can provide significant accurate results in detecting or even discriminating the latent faults. However, it is just a manner of supervised learning that implies the neglect of large amounts of unlabeled sensor data, while performing unsatisfactorily when labeled sensor data are insufficient, which is often the case in chemical systems. Furthermore, two weaknesses must be improved for better diagnosis:

(a) The poor ability of learning complex nonlinear relationships because of such a shallow architecture [17];
(b) The features are only extracted based on signal-based techniques, and the model performance strongly depend on expert and a priori knowledge, which have significant limitations in terms of applicability.

Deep learning is a type of semi-supervised learning that holds significant strengths in overcoming the aforementioned weaknesses in current knowledge-based methods through multiple non-linear transformations and approximate non-linear functions for various diagnosis issues. Deep learning demonstrates significant ability in data expression compared to shallow learning and is able to learn the feature of input patterns adaptively for intelligent fault diagnosis rather than just depending on manual extraction. Hinton et al. [18] proposed an unsupervised learning algorithm that trains the deep belief network through the manner of greedy layer-by-layer. The employment of this deep learning training algorithm solves the training problem of deep neural network(DNN) that easily incur leading to catastrophic failure [17,19]. This development promotes the move of deep learning technique into a new platform, and motivates significant performance in image, face recognition and natural language processing [20,21,22]. Deep learning also has significant merit in a few current studies on fault diagnosis using large amount of data, mainly in machinery [9,23,24,25]. However, the preponderance of deep learning also experiences the practical problem of how to select the sensor data collected to be labeled, that is, further improvement in the effective utilization of labeled sensor data is still needed since a model needs a priori knowledge of data-self, to perform better, whereas the labeling of collecting sensor data can be expensive and difficult in chemical industry systems. Several studies have shown that labeling requires a large number of experts and more than 10-fold that of time consumed relative to obtaining data [26]. 

The objective of active learning is to learn a function that improves the model while requiring as little sensor data labeling as possible. Active learning has been investigated for many real world problems, such as image classification [27,28], biomedicine [29,30], and system monitoring [31,32], which have presented a comprehensive survey. However, the combinations of deep learning and active learning, which integrates the merit of feature representation and data efficiency, have not been employed in current chemical fault diagnosis research. It is desirable to employ active learning to rank the most informative sensor data to be labeled and deep learning to fine-tune the model in an active manner and thus minimizes the number of training samples necessary to optimize the discrimination capabilities of the fault diagnosis model as far as possible. 

This study presents a novel DNN with active learning using large amount of chemical sensor data for chemical fault diagnosis. It is a combination of deep learning and a novel active learning criterion that targets the difficulty of consecutive fault diagnosis in chemical systems. The initial DNN model employs a deep architecture with stacked denoising auto-encoder (SDAE) and works through a hierarchical successive learning process where deep learning technique is applied for the feature representation of diagnosis sensor data. The features learned are added to the top Softmax regression layer to construct the discriminative fault characteristics for diagnosis in a supervised manner. For further fine-tuning of the DNN, in contrast to the available methods, we select the most useful samples by the combination of the proposed criterion Best vs. Second Best (BvSB) and Lowest False Positive (LFP), which improve DNN actively for labeling through a novel active learning criterion to target the chemical sensor data, which has better improvement in accuracy and false positive rate than existing methods. 

Compared with the existing related methods, the contributions of the proposed method are summarized as follows:

(1) Deep learning is able to learn the feature of diagnosis sensor data adaptively for intelligent fault diagnosis rather than merely relying on manual extraction.
(2) The method performed excellently in obtaining the potential information and fault characteristics of raw sensor data by multiple non-linear transformations and approximate non-linear functions and presented higher diagnosis accuracy than methods based on shallow architecture. Therefore, the proposed method is a preferred approach for diagnosis in complex chemical systems.
(3) The combination of deep learning and active learning is proposed in the chemical fault diagnosis, which improves the existing diagnosis methods significantly. Compared with available active learning methods, a novel active learning criterion combined with BvSB and LFP is presented, which is an active labeling method for the cost-effective selection of chemical sensor data to be labeled and achieves the selection of the most valuable samples for inducing the DNN in chemical fault diagnosis, thus improving the model performance maximally.

The remainder of this paper is organized as follows: The applicability analysis and preparations related to the proposed method are formally introduced in Section 2; detailed descriptions of the proposed method are presented in Section 3; the simulation evaluation is provided in Section 4; and the conclusions are presented in Section 5.

## 2. Applicability Analysis and Model Preparation

We first present the merit and applicability of deep learning with chemical sensor data for fault diagnosis in complex chemical processes in this section. Subsequently, we present several details about the model preparation by presenting an overview of the sparse auto-encoder and the method of data preprocess involved in the construction of DNN. 

### 2.1. Advantage of Deep Learning with Chemical Sensor Data 

The aim of machine learning is to learn knowledge from data for application through specific algorithms. Mining the discriminative feature concealed in the data is a prerequisite, that is, an abstract concept that is provided for classification or recognition. Feature selection is a major part of machine learning that requires a considerable investment of resources in particular areas, including fault diagnosis [9]. Deep learning is a method that can adaptively mine the feature from raw sensor data, namely, transforming original sensor data into a highly abstract expression through the stack of some nonlinear models. With adequate transformation, deep learning attempts to find the internal structure of input and potential relationship between variables [33]. 

The majority of traditional models, such as SVM, MLP, and radial basis function (RBF), are considered as shallow architectures that have less than three layers of computation units [23]. Recent theories have shown the difficulty of maintaining the representational ability in the case of reducing the algorithm structure, that is, the networks with an inadequate depth of layers are deficient in representing and providing limitations on several learning tasks [17,34]. Deep learning constructs a deep model through the simulation of the learning process of the human brain. In the field of chemical fault diagnosis, deep learning obtains potential information and the fault characteristics of original input of chemical sensor data through multiple non-linear transformations and approximate non-linear functions with a small error that is attributed to various diagnosis issues [9]. Especially, the application of greedy layer-by-layer training algorithm solved the training problems of deep hierarchical structures that may easily stuck into poor local optima [18]. This development improved the performance of feature extraction and identification of health condition, which led to the applicability of deep learning in chemical fault diagnosis. Chemical systems are commonly composed of different kinds of sub-systems that involve multidimensional heterogeneous sensory signal and highly nonlinear correlations between diagnosis sensor data and the results. Furthermore, some biochemical reactions vary highly in various configuration of constitutes. Accordingly, the combination of deep learning and chemical fault diagnosis is applicable and significant. 

Most of the collecting chemical sensor data are unlabeled data that are ignored in traditional supervised models like ANN and SVM. Furthermore, the number of quality sensor data is much less than that of the processing data because of the disparity in sampling rate [17]. Therefore, only a small number of chemical sensor data can considered, and the rest of the process samples containing abundant information are ignored. Deep learning is semi-supervised learning approach that applied these sensor data on unsupervised feature extraction. The chemical sensor data abandoned by previous methods can be used for unsupervised pre-training to extract explicit latent variables. It is therefore plausible that more diagnosis models that are significant may be attained with more sensor data used. Accordingly, attempting to apply the deep learning technique to chemical fault diagnosis is worth the effort.

### 2.2. Sparse Auto-Encoder

**Figure 1:**
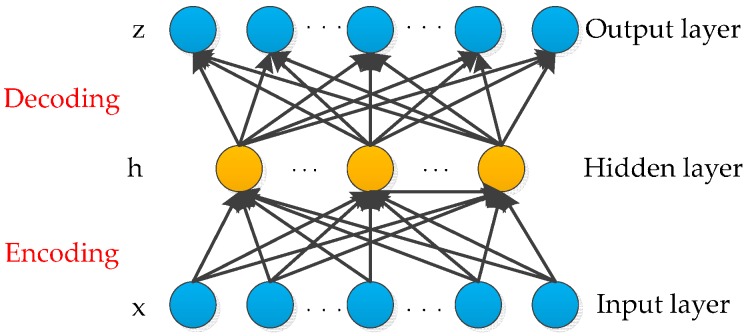
Auto-encoder.

Auto-encoder is a symmetrical neural network that learns the feature of an input in an unsupervised manner. The auto-encoder is composed of the input layer, hidden layer and output layer [35]. The basic structure of an auto-encoder is shown in Figure 1. The hidden layer encodes the input, whereas the output layer reconstructs the input by minimizing the reconstruction errors to obtain the best expression of data.

From the measured chemical sensor data for diagnosis, the unlabeled sensor dataset can be represented as $X=\{x_{1},x_{2},x_{3}...x_{n}\},x_{i}\in {ℜ}^{n}$, where *n* is the number of samples for each diagnosis input. In the phase of encoder network, the encoder transforms the input vector $x_{i}$ to a hidden representation h by an encoding function denoted by $f_{\theta }$:

(1) $$h(x_{i})=f_{\theta }(x_{i})=f(W_{1}x_{i}+b_{1})$$

where $f_{\theta }$ is a nonlinear active function and $\theta =\{W_{1},b_{1}\}$. $W_{1}\in {ℜ}^{D_{i}\times D_{h}}$ is the weight matrix of the encoder and $b_{1}\in {ℜ}^{D_{h}}$ is the bias. The number of units in the input the hidden layers are denoted by $D_{i}$ and $D_{h}$, respectively.

In the phase of decoder network, a reconstruction function denoted by $g_{\theta^{\prime }}$ maps h back into the input, namely producing a reconstruction *z*:

(2) $$z_{i}=g_{\theta^{\prime }}\left(h(x_{i})\right)=W_{2}h(x_{i})+b_{2}$$

where $\theta^{\prime }=\{W_{2},b_{2}\}$, and $W_{2}\in {ℜ}^{D_{h}\times D_{i}}$ and $b_{2}\in {ℜ}^{D_{i}}$ are the weight matrix of the decoder and bias, respectively. 

In the model learning perspective, the weights of encoder and decoder are learned simultaneously during the reconstruction of the original sensor data as long as possible, that is, attempting to incur the minimization of the loss function that denotes the discrepancy between the input signal *x* and reconstruction *z*. For dataset $\left\{(x^{1},y^{1}),...,(x^{n},y^{n})\right\}$, where $x^{i}=y^{i}$ in the auto-encoder, the loss function can be represented as:

(3) $$J(W,b)=[\frac{1}{n}\sum_{i=1}^{n}(\frac{1}{2}{\left\|z_{i}-y_{i}\right\|}^{2})]+\frac{\lambda }{2}\sum_{l=1}^{m_{l}-1}\sum_{i=1}^{s_{l}}\sum_{j=1}^{s_{l+1}}{\left(W_{ij}^{\left(l\right)}\right)}^{2}$$

where the first part is the mean square variance, the second part is the regularization term that reduces the range of weights and prevents over fitting, and λ is the weight of regularization term. 

The sparse penalty term is added to the auto-encoder to obtain the complex nonlinear relationship between features, such that the learned features are of the sparse constraint that captures the most significant factor of the input patterns [36]. We will minimize the loss function with a sparse to achieve the sparse representation, as follows:

(4) $$J_{sparse}(W,b)=J(W,b)+\beta \sum_{j=1}^{s_{2}}KL(\rho \|{\hat{\rho }}_{j})$$

where β is the weight of sparsity term; $s_{2}$ is the number of units in the hidden layer; ρ is a sparsity parameter and is typically a small value close to zero; and ${\hat{\rho }}_{j}$ is the output average in the hidden layer. KL(.) is the Kullback–Leibler divergence (*KL* divergence), which denotes the relative entropy between ρ and ${\hat{\rho }}_{j}$ [37]. KL(.) is a convex function that possesses the property KL(.) = 0 if ${\hat{\rho }}_{j}=\rho$ and increases monotonically as ${\hat{\rho }}_{j}$ approaches ρ. KL(.) acts as the sparsity constraint on the coding that is expressed as:

(5) $$KL(\rho \|{\hat{\rho }}_{j})=\rho \log\frac{\rho }{{\hat{\rho }}_{j}}+(1-\rho )\log\frac{1-\rho }{1-{\hat{\rho }}_{j}}$$

The optimal parameters of the sparse auto-encoder *W* and bias *b* can be solved by minimizing the loss function with sparsity constraint. The optimization process can be realized using the back-propagation (BP) algorithm [38]. For an auto-encoder, the output of the hidden layer determines the potential expression of the input sensor data, and limits the ability of representation significantly because of the shallow architecture. Stacked auto-encoder attempts to mine the characteristics of the input patterns in unsupervised manner through a stack of auto-encoders, which presents a superior effect on feature learning.

### 2.3. Method of Data Preprocessing in Models

Data preprocessing is the foundation for the preeminent performance of models, especially the feature normalization that standardizes the range of values stored in different features [7]. In the field of chemical fault diagnosis, different indices of sensor data possess dimensions, whereas sensor measurements may have different units that lead to diverse scales. For example, revolving speed has “rpm” as its unit, whereas displacement is measured in the unit of “mm”. Accordingly, the influence of different scales between process variables using normalization should be avoided. We adopt the Z-score normalization in this study.

Z-score normalization is a data preprocessing approach based on the mean and standard deviation of raw data. It has become a traditional approach in the field of fault diagnosis and health monitoring, which take advantage of not knowing the maximum and minimum of attributes beforehand and the significant effect of reducing the effect of noise [39]. The details of Z-score normalization are as follows. 

For the sensor dataset denoted by matrix $X\in {ℜ}^{m\times n}$, *m* is the number of samples and *n* is the number of attributes. Z-score method normalizes X to a dimensionless matrix with zero mean and unit variance. The $i^{th}$ row of the processed data can be computed by:

(6) $$X_{i}^{*}=\frac{X_{i}-\bar{x}}{\sigma },\;for\;i\in \{1,2...m\}$$

where $X_{i}$ is the sample of sensor dataset X, $\bar{x}$ is the mean vector of the original data and σ is the standard deviation vector correspondingly.

## 3. A Fault Diagnosis Method with Active Deep Network

**Figure 2:**
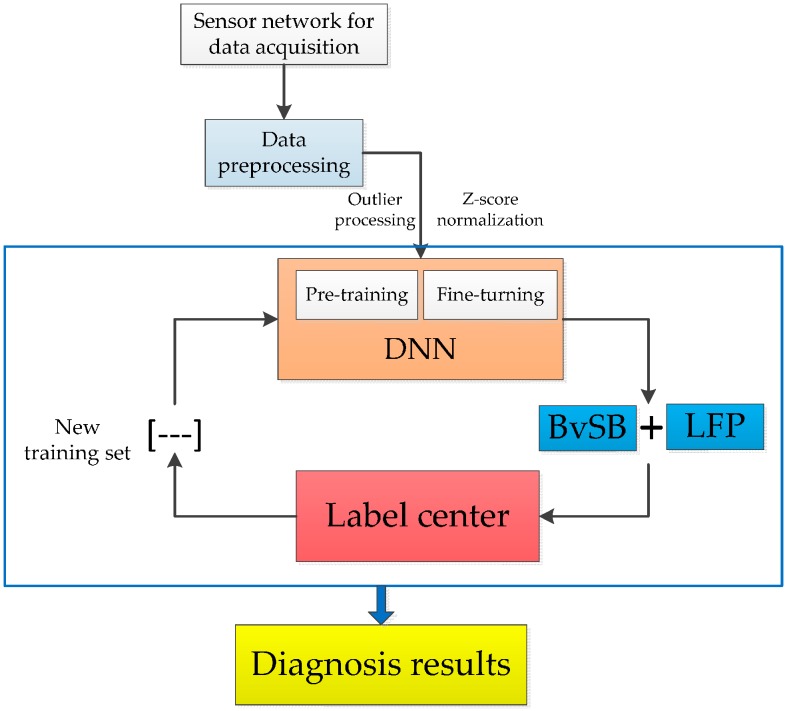
An illustration of proposed method for chemical fault diagnosis.

We propose a chemical fault diagnosis method with active DNN in this section. DNN is utilized in this method to excavate potential information on chemical sensor data and is combined with a novel active learning criterion to obtain fault diagnosis in an effective manner. The general framework of the proposed method is indicated in Figure 2. We present the description of the proposed method in following part.

### 3.1. Unsupervised Learning Using SDAE 

The auto-encoder without any constraint is prone to copy the input to the output directly. The model exhibits poor performance with greater reconstruction error, especially when the difference between the training and test data is predominant. Denoising auto-encoder attempts to make the learned feature representation robust rather than simply repeating input by adding partial corruption to the input pattern, which can be employed to train the stacked auto-encoder to initialize the deep architecture [24]. SDAE achieves the highly abstract expression of the original chemical sensor data through the stack of multiple denoising auto-encoders. The process can be reformulated with more detail as follows:

First, random noise is added into the original input via a random map: $\tilde{x}\to q(\tilde{x}|x)$ and is mapped into the hidden layer:

(7) $$h=f_{\theta }({\tilde{x}}_{i})=f(W_{1}{\tilde{x}}_{i}+b_{1})$$

The activation function can be represented as:

(8) f(a)=1/(1+exp(-a))

The reconstruction of the input pattern is represented in same manner as the spare auto-encoder:

(9) $$z_{i}=g_{\theta^{\prime }}\left(h\right)=W_{2}h+b_{2}$$

The reconstruction error is computed from the difference between $x_{i}$ and $z_{i}$, and can be minimized by solving the cost function (Equation (4)). The training epoch will be repeated until the value of the cost function goes lower than a pre-set threshold, which is close to zero. By this time, the parameter of the denoising auto-encoder $\left\{\theta ,\;\theta^{\prime }\right\}$ can be obtained. Considering that the SDAE is constructed with a stack of multilayer denoising auto-encoders. The weights and bias matrices of the SDAE are expressed by $W_{all}$ and $b_{all}$, respectively. The parameters of SDAE can be computed by:

(10) $$W_{all},b_{all}=\underset{\theta_{i},\;\theta_{i}^{\prime }}{\arg\;\min}\;J_{sparse}(W,b)$$

where i=1...n, and *n* represent the number of layer about the constructed SDAE.

**Figure 3:**
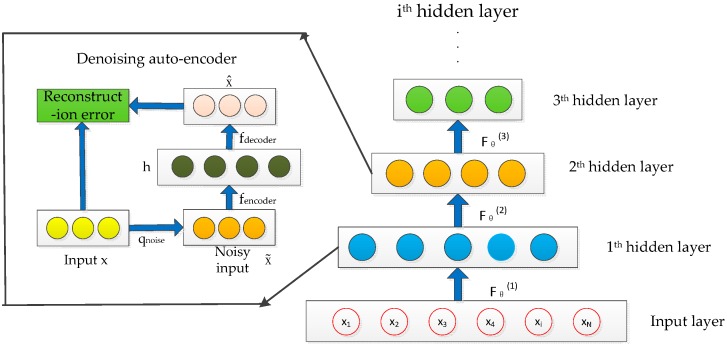
Stacked denoising auto-encoder (SDAE).

The SDAE process is shown in Figure 3. The trained parameters of the auto-encoder are used to initialize the parameters of the first hidden layer of the SDAE, and the first hidden layer is trained by the input pattern, and then the output of the first hidden layer is used as the input of the second hidden layer. The training steps are repeated in sequence until the final auto-encoder is trained and the ideal data expression is completed. The DNN with SDAE pre-training can be described as: For l∈{1,...,L−1}:

(11) $$h_{l+1}=f(W_{l+1}h_{l}+b_{l+1})$$

where $h_{l}$ represents the output vector of the l layer, $W_{l}$ is the weight matrixes, $b_{l}$ is the encoder bias vector, f(x) is the activation function used in the encoder, and $h_{L}$ is the final output features. 

### 3.2. Supervised Learning Stage and Fine-Tuning

**Figure 4:**
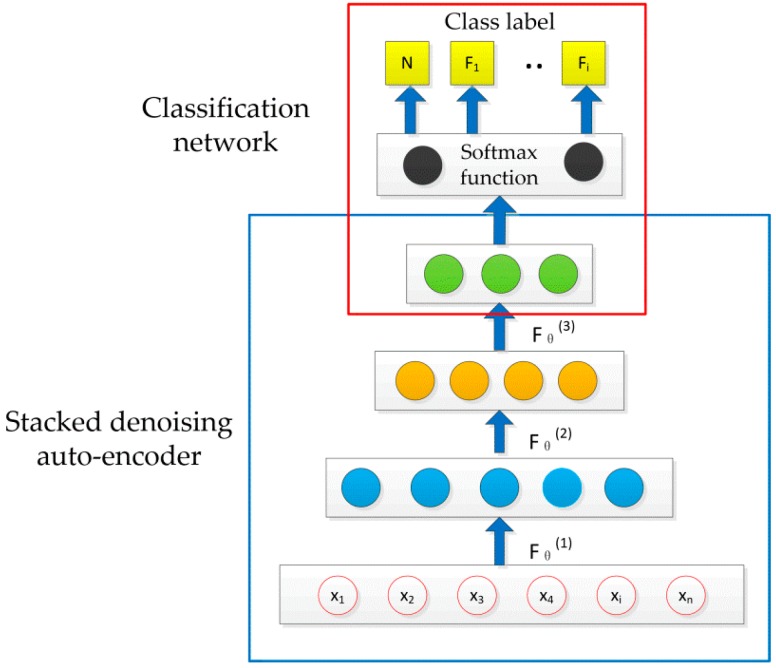
Deep neural network (DNN) framework.

After the completion of the unsupervised pre-training phase through the greedy layer-by-layer approach, fine-tuning is utilized in the next step of the DNN training by adding a Softmax classifier on top of the network as shown in Figure 4. For the random layer l∈{1,...,L−1} in DNN, the weights of the $l^{th}$ layer are the same as that of the SDAE, and the weights of top network are initialized randomly. The fine-tuning process is a stage of supervised learning. The outputs of DNN can be expressed as:

(12) $$h_{L+1}=P(W_{L+1}h_{L}+b_{L+1})$$

where P(.) is the predictive function. Finally, BP algorithm is applied to train the entire deep network and can be described as:

(13) $$\theta^{*}=\underset{\theta }{\arg\;\min}\sum_{i=1}^{N}L(F_{\theta }(C_{i}),T_{i})$$

where θ are the model parameters $\left\{W_{l},b_{l}\right\}$, l∈{1,...,L+1}, L(X,Y) is the cost function of the entire network, and F(C) is the compound function of the DNN, when the *T* is the objective element:

(14) $$F(C)=P_{\theta_{L+1}}(f_{\theta_{L}}(...f_{\theta_{l}}(...f_{\theta_{1}}(C))))$$

More information regarding the training process of the BP algorithm can be found in [38].

**Figure 5:**
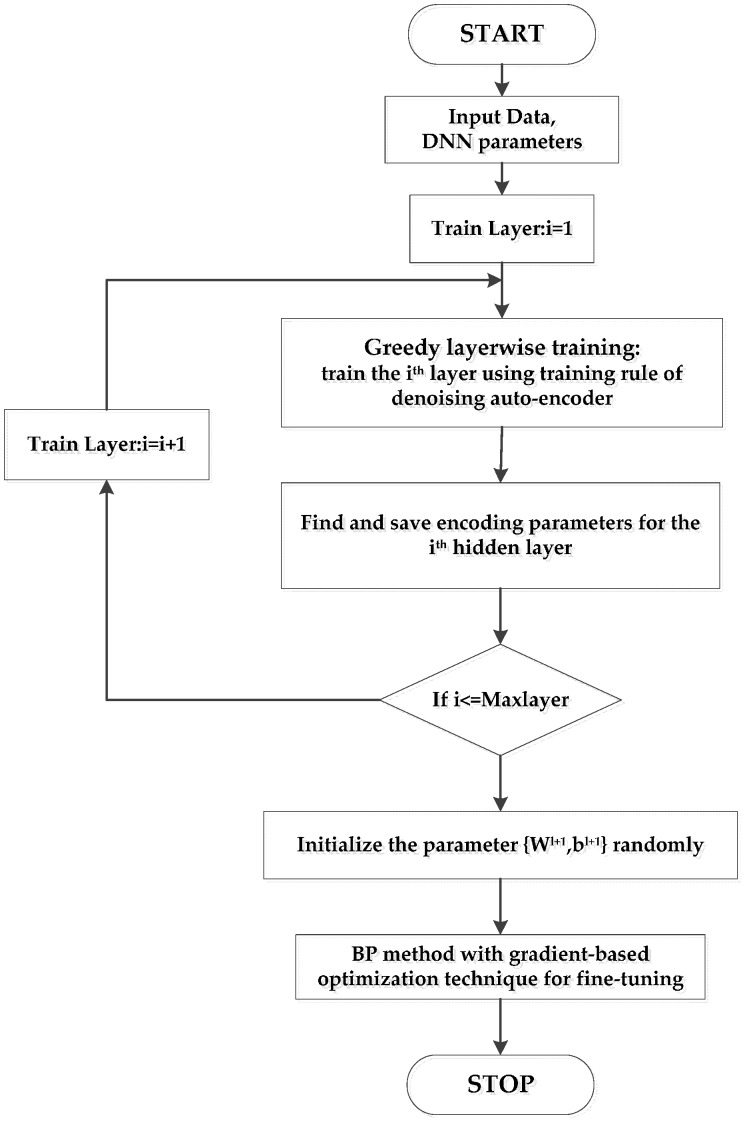
Procedure of DNN training process.

The supervised learning stage attempts to utilize a small amount of labeled sensor data to reduce the training error further and improve the classification performance of DNN. The main problem of DNN training about poor local optima, which can cause catastrophic failure, is successfully addressed by the combination of the greedy layer-by-layer pre-training and supervised fine-tuning [34]. This semi-supervised learning realizes the effective application of unlabeled sensor data, which occupies a large proportion of chemical big data. The overall training procedure of DNN is shown in Figure 5.

### 3.3. Active Learning Procedure for DNN (AL-DNN)

The acquisition of labeled sensor data in the application of chemical fault diagnosis is expensive and time consuming because of the high complexity of industrial systems and the variety of fault types, which require a lot of resources and manpower, such as seeking for an expert mark [29,32]. In contrast, unlabeled sensor data can be effectively collected by deploying a sensor network [4,5]. Therefore, exploring a method to productively use the labeled sensor data in the field of chemical fault diagnosis is particularly crucial. This study presents an active learning method, which is applied to DNN based on SDAE for further fine-tuning. The proposed active learning method is a newly active labeling method for the cost-effective selection of collecting sensor data to be labeled and improves the model performance maximally. 

The labeled sensor dataset to be diagnosed is denoted by $L=\{x_{1},x_{2},...,x_{n}\}$ and the labels by $Y_{i}=\{y_{1},y_{2},...,y_{n}\}$, where $x_{i}$ is the sample and $y_{i}$ is the label of the $i^{th}$ sample. The unlabeled sensor dataset can be expressed by $U=\{x_{n+1},x_{n+2},...,x_{n+m}\}$. We assume the independent identical distribution of each sample and the label of each sample is decided by a conditional distribution P(y|x). First, DNN is trained by the unlabeled sensor dataset *U* and initial labeled sensor dataset *L*. Active learning allows us to rank the final sensor dataset $D_{test}=\{x_{s_{1}},x_{s_{2}},...,x_{s_{k}}\}\subseteq U$ for expert labeling, thus minimizing the number of training samples necessary to maintain the discrimination capabilities as high as possible. The most ambiguous chemical sensor data are provided to the expert for labeling and then used to retrain the classifier. 

The posterior probabilities of associating a sample to a given class $\rho_{\Theta }(y_{i}|x_{i})$ were selected as the measure index of model uncertainty in our study. This work combines two different selection criteria to select the most relevant samples during the each iteration:

Best vs. Second Best criterion (BvSB): the samples that having the lowest difference between the two highest posterior probabilities.

(15) $$x_{BvSB}=\underset{x}{\arg\;\min}(\rho_{\Theta }(y_{1}|x)-\rho_{\Theta }(y_{2}|x))$$

where $\rho_{\Theta }(y_{1}|x)$ and $\rho_{\Theta }(y_{2}|x)$ are the two highest posterior probabilities corresponding to the DNN outputs.

Lowest False Positive criterion (LFP): under the condition of the exception class by model outputs, the samples that have the lowest difference in posterior probabilities between this exception class and the corresponding normal class.

(16) $$x_{LFP}=\underset{x}{\arg\;\min}(\max(\rho_{\Theta }(y_{F}|x))-\rho_{\Theta }(y_{N}|x))$$

where $\rho_{\Theta }(y_{F}|x)$ is the posterior probabilities under the condition of the exception class by model outputs and $\rho_{\Theta }(y_{N}|x)$ is the posterior probabilities corresponding to the normal class.

*k* samples were selected for final fine-tuning by the integration of the two criteria that can be described as:

(17) $$X_{k}=x_{BvSB}\cup x_{LFP}$$

For the BvSB criterion, the difference between the two highest posterior probabilities is indicative of the manner in which a sample is shown and the uncertainty of classifier. The classifier confidence is low when the two highest values are close. In the second criterion, the selected samples are most probably prone to false positives, which result in fault missing. It is worth noting that the running with failure for a long time results in potent damage in industrial systems [3]. Thus, the damage of false positive in chemical fault diagnosis is far greater than the other justifications, such as miscarriage. The following Algorithm 1 provides the main steps of the proposed method called AL-DNN.

**Algorithm 1: AL-DNN****Input:**
Labeled sensor dataset: $L=\{x_{1},x_{2},...,x_{n}\}$Unlabeled sensor dataset: $U=\{x_{n+1},x_{n+2},...,x_{n+m}\}$Parameters of DNN: the number of hidden layer n; learning rate λIterations: ITERNumber of chosen samples at the each iteration: *k* **Output:**
The result of fault diagnosis **Main step:**
Data preprocessing: data normalization by (Equation (6))Pre-training (unsupervised): Use all samples in *U* to train a SDAE layer-by-layer, compute the weights of SDAE by minimizing the cost Function (Equation (10)): $W_{all}$, $b_{all}$Use $W_{all}$ and $b_{all}$ to initialize DNN, Use labeled sensor dataset L to fine-tune DNNObtain the overall parameters of trained DNN: $\theta^{*}$Classify all unlabeled samples in *U* using the constructed DNN: f(U)=Test(C,U)Initialization: $D_{test}$ = ∅Active learning stage:
For iteration = 1: ITER
Compute the posterior probabilities of all unlabeled samples in *U*: *post*Select the samples having the lowest difference between the two highest values in *post* by (Equation (15)): $$x_{BvSB}=select\_BvSB(U)$$Select the samples that will most probably show the false positive using (Equation (16)): $$x_{LFP}=select\_LFP(U)$$Obtain k chosen samples $X_{k}=\{x_{s1},x_{s2}...x_{sk}\}$ from *U* by the combination of two criteria: $$X_{k}=x_{BvSB}\cup x_{LFP}$$Ask an expert to label the sensor dataset $X_{k}$: $label(X_{k})$Update the unlabeled sensor dataset: $U\leftarrow U-X_{k}$Augment the active training set: $D_{test}\leftarrow D_{test}\cup X_{k}$Update the weights of DNN by fine-tuning using $D_{test}$ **End**

## 4. Experimental Study

This section presents the development of the chemical fault diagnosis based on the proposed method in two diagnosis cases. The University of California Irvine(UCI) dataset is employed to evaluate the capability and superiority of the proposed framework compared with other framework in the viewpoint of general situation and we further validate the method in Tennessee Eastman (TE) dataset that for a chemical process. Its performance is compared with other related methods. 

### 4.1. Data Description

#### 4.1.1. Case Study 1: UCI Dataset—Dataset for Sensorless Drive Diagnosis Dataset

The experimental data used here are from the UCI machine learning repository, and are provided by a real sensor signal. We employed the proposed method to validate the superiority of active DNN framework in model performance. In the dataset, features are extracted from electric current drive signals by empirical mode decomposition (EMD). The drive has intact and defective components. This results in 11 different classes with different conditions. Each condition measured several times using 12 different operating conditions, that is, by different speeds, load moments and load forces. The current signals are measured with a current probe and an oscilloscope on two phases. Six classes of the current conditions are used in this study to test the performance of the proposed method. Type A corresponds to the normal condition whereas B-F corresponds to the different fault types caused by defective components. A total of 5319 samples for each health condition were used. Twenty thousand samples were selected as unlabeled data and used in pre-training, whereas 500 samples were selected as initial labeled data for fine-tuning. The other samples were selected as test data. 

#### 4.1.2. Case Study 2: TE Dataset—Dataset for Tennessee Eastman Process (TEP)

**Figure 6:**
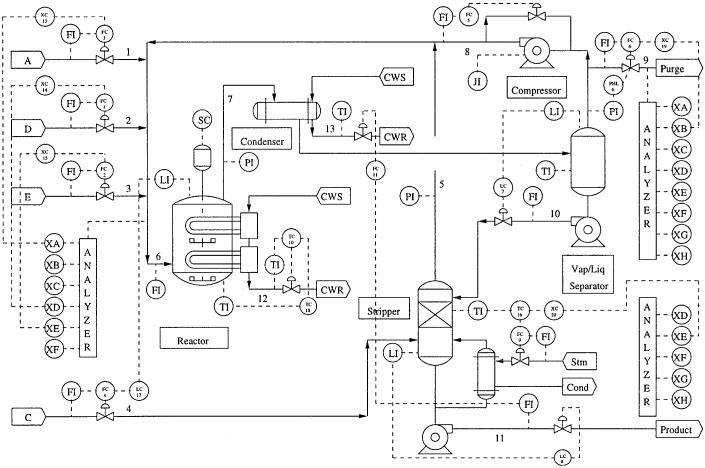
Flow diagram of the Tennessee Eastman process (TEP).

TEP is a benchmark simulation model that tests the fault diagnosis approaches of process control in real chemical processes [40]. TEP has five major units: a reactor, a compressor, a separator, a stripper and a condenser. TEP involves large amount of chemical sensor data that can provide fault diagnosis. Many variables have strong correlations and coupling between each other (including 41 measurement variables and 11 effective control variables). Therefore, TEP is a highly complex nonlinear process involving multidimensional heterogeneous sensory signal and highly nonlinear correlation between processing variables. The flow diagram of the process is shown in Figure 6. 

The experimental dataset is generated by the TEP simulation model, and 21 types of faults can be simulated. The simulation times of the training and the test sets are 24 h and 48 h, and the faults appear after 1 h and 8 h, respectively. In this study, six classes of known faults and an unknown fault (IDV17 of the dataset) are used for the experiment, labeled 1–7, respectively. Thus, the dataset includes 8820 samples; 100 samples were selected as the initial labeled data for fine-tuning and 2000 samples for testing. The other samples were selected as unlabeled data for pre-training. The specific description of fault in the experiment is indicated in Table 1.

### 4.2. Experiment Setup

In this study, DNN has four layers that are designed using cross validation. The unit number of the input layer is determined by the dimension of the diagnosis input, whereas the unit number of the output layer is determined by the number of fault states. For the two experimental datasets, the numbers of units in the hidden layer are set to {200,100} and {100,50}, respectively. The activation function of the DNN is sigmoid. Sparsity penalty was adopted in the DNN for the two datasets and is an effective method to prevent over fitting as well as improve the generalization ability of the DNN. The details of the DNN parameters for the two datasets are shown in Table 2. In addition, the training needs to be repeated several times for stable and reliable results because of the inevitable randomness of the neural network. In the experiment, the results are obtained after training 10 times repeatedly.

### 4.3. Result and Analysis in Different Diagnosis Cases

#### 4.3.1. Result on UCI Dataset 

**Figure 7:**
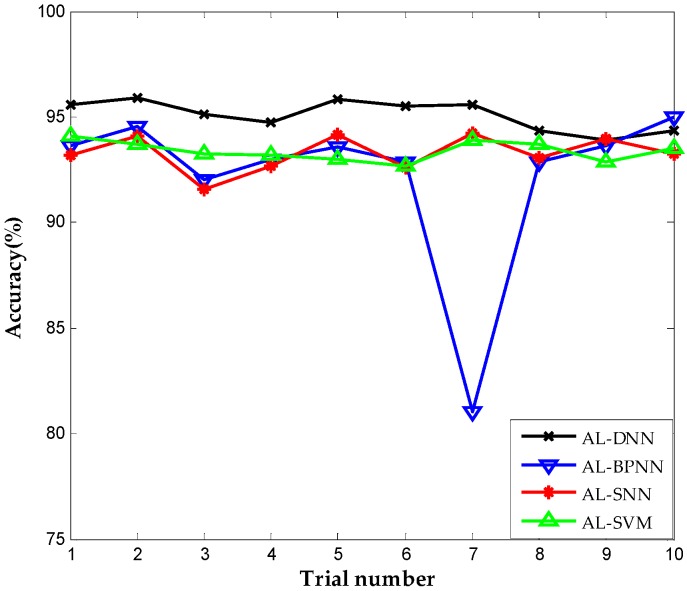
Correct diagnosis rate of active learning for deep neural network (AL-DNN), active learning for back propagation neural network (AL-BPNN), active learning for single neural network (AL-SNN), and active learning for support vector machine (AL-SVM).

Shallow architecture accounted for most parts in the current fault diagnosis for chemical industry. Two widely used algorithms in fault diagnosis that have shallow architectures, namely, neural network with single hidden layer (SNN) and support vector machine (SVM) are employed with same active learning criterion for comparison to verify the superiority of proposed approach. We try to validate the merit about the deep architecture and the employment of deep learning in DNN, so we take the above shallow architecture to the same dataset. Meanwhile, the back propagation neural network (BPNN) that shares the same architecture and trained by the same parameters with DNN is included in the comparison to further demonstrate the performance of deep learning in the construction of DNN, since BPNN lacks feature learning in the pre-training stage relative to the proposed method. We call the above methods active learning for single neural network (AL-SNN), active learning for support vector machine (AL-SVM), and active learning for back propagation neural network (AL-BPNN), respectively. Figure 7 shows the diagnostic accuracies using different methods in the same iteration number of active learning, that is, the same number of labeled sensor data for training. Ten trials are carried out repeatedly, and we clearly notice that all the accuracies using the proposed method are nearly stable at 95%, while the SNN-, SVM- and BPNN-based methods have lower diagnosis accuracies. It presents the ability to obtain more abundant information using the proposed method than the approach with only shallow architecture. This advantage becomes more evident in the complex chemical industry, which has numerous variables and a highly nonlinear relationship.

**Figure 8:**
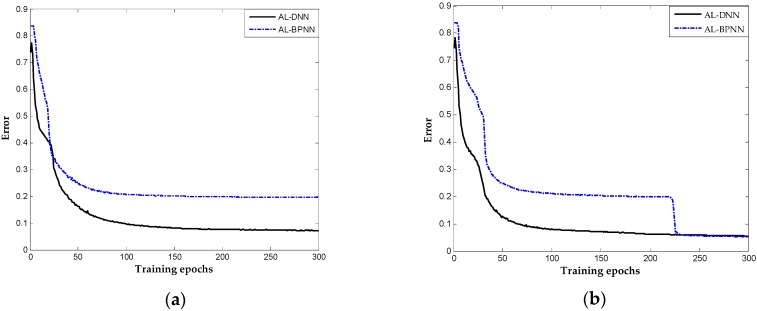
Curves of error of the proposed and AL-BPNN methods: (**a**) the seventh trial of dataset; and (**b**) the ninth trial of dataset.

It is worth mentioning that the performance in the tenth trial using BPNN-based method was good while performing unsatisfactorily in presenting large result fluctuations in different trails. In trials, BPNN-based method failed catastrophically as it is a deep architecture for random initial parameters. Taking the seventh and ninth trials as examples, the errors of these trials in the different number of epochs are indicated in Figure 8a,b. The results show that the proposed method achieve satisfactory results after approximately 100 iterations and updated smoothly into significant solutions in the two trials, whereas the BPNN-based method presents a slower convergence rate. In the seventh trial, the BPNN-based method falls into the local optimal solution where the diagnosis error is approximately 0.3. Neural networks with deep architecture have a significant capability of distinguishing the highly complex characteristics of industry system, but not in a stable manner because of random factors in model construction that cause the training to fail catastrophically. The result indicates that the proposed method is more robust and stable, which overcome the training problem of DNN and is more effective in achieving better diagnosis results in chemical occasions.

**Figure 9:**
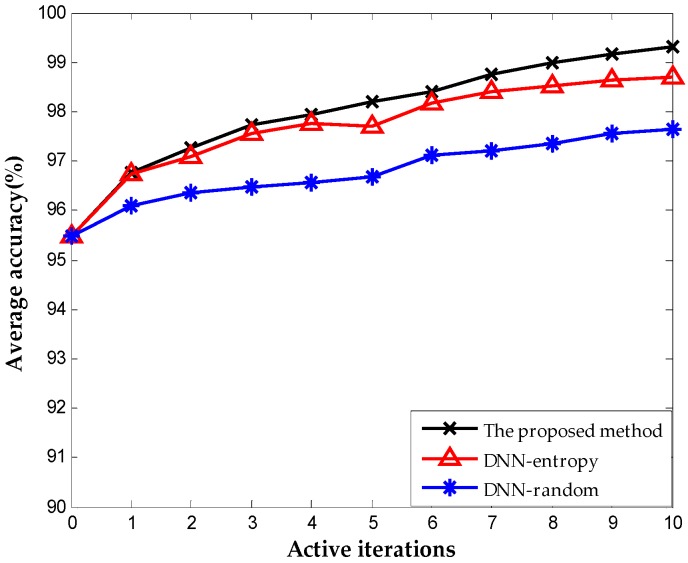
Classification accuracy during each iteration.

**Figure 10:**
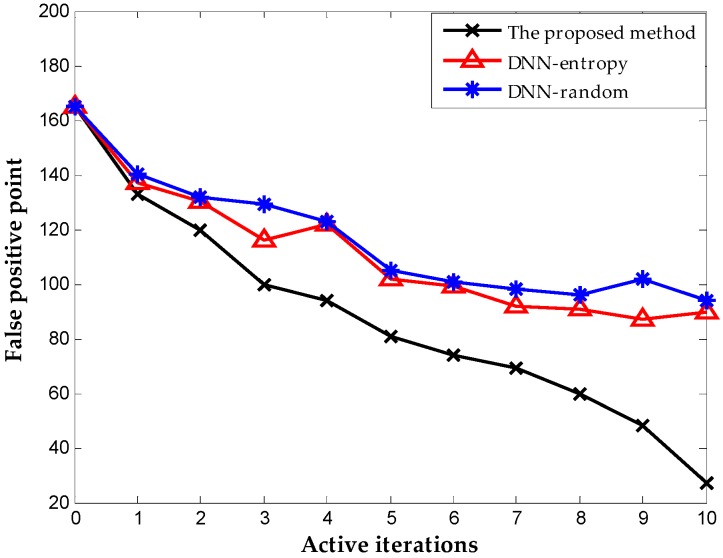
The number of false-positive point during each iteration.

We include the results obtained by random selection and entropy selection criteria in DNN for comparison, which are referred to as DNN-random and DNN-entropy, respectively, to highlight the benefit of using the proposed active learning criterion. The number of samples for fine-tuning is 500 initially. We update the weights of DNN by fine-tuning 50 new samples obtained by different selection criteria. Figure 9 shows the behavior of diagnosis accuracy during the each iteration. The proposed method and DNN-entropy clearly provide better results than DNN-random, which ignores active learning, and the diagnosis performance improves by approximately 2%. This improvement implies the effectiveness and significance of active learning in chemical fault diagnosis. It is worth noting that the proposed method outperforms DNN-entropy in with minor superiority. It is a presentation that the proposed method provides better applicability and adaptability with less sensor data than other active learning criteria in the chemical industry. Furthermore, the comparison of the number of false positive point of three methods is indicated in Figure 10. The result shows that the proposed method significantly improves the false positive to a great extent and presents a better effect with the increase of iterations. This excellent performance in fault detection is always a key problem in system maintenance and monitoring. Even though DNN-entropy is effective in improving model accuracy, the effect on the enhancing omission of fault is limited. In industrial systems, a missing fault means the fault operation of systems for a long time, where the damage is greater than other diagnosis states, especially in chemical processes. Accordingly, the proposed method has a significant appeal and potential for fault diagnosis and system monitoring in the chemical industry.

**Figure 11:**
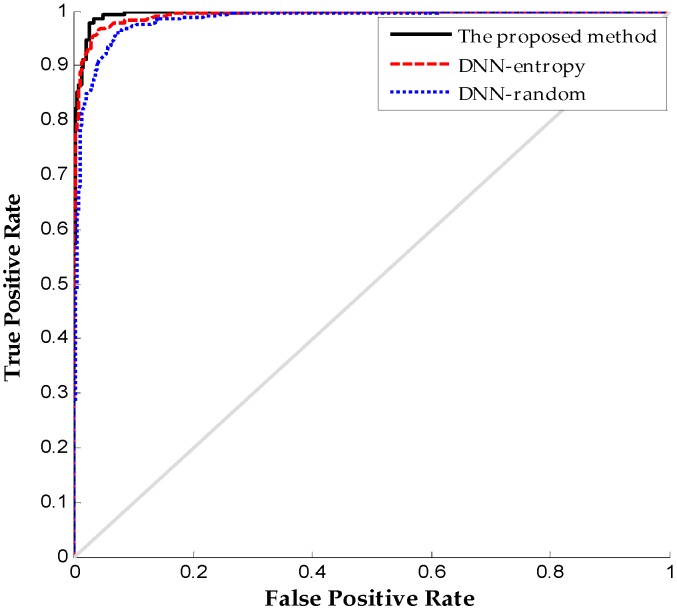
ROC (receiver operating characteristic curve) results comparison on different method.

Figure 11 shows the ROC (receiver operating characteristic curve) curve comparison on different labeled sensor data to further indicate the superiority of the proposed method in fault diagnosis. The ROC curve reflects the relationship between the true positive and false positive at different thresholds. The ROC curve shows that the proposed method has a larger area under the curve than DNN-random and DNN-entropy. Consequently, the proposed method provides the best performance through a novel active learning criterion for fault diagnosis. The point under this ROC curve is the optimal threshold with the least error, which has the least numbers of false positives and false negatives.

#### 4.3.2. Result on TE Dataset

**Figure 12:**
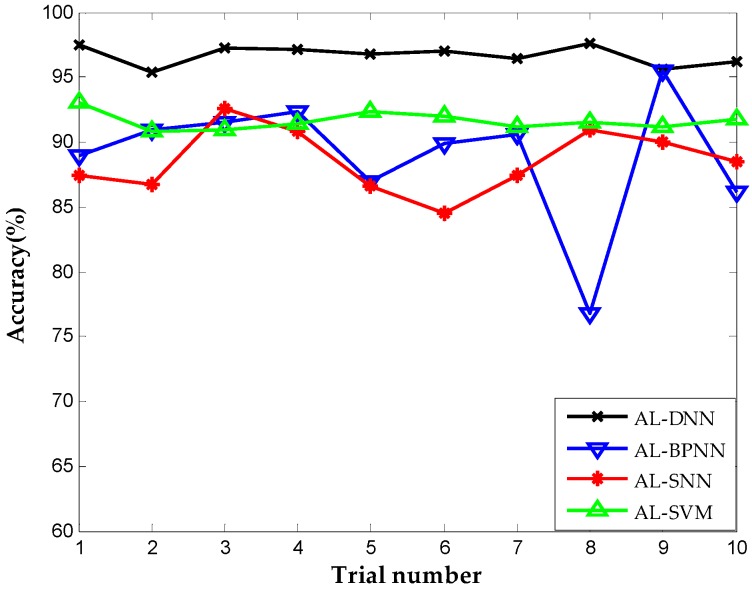
Correct diagnosis rate of AL-DNN, AL-BPNN, AL-SNN, AL-SVM on TE dataset.

We repeat the above experiments on the TE dataset to further assess the generalizability and applicability of proposed method. It is worth mentioning that the methods for comparison are identical to those in the above experiment. The detailed classification result using different diagnosis approaches with 100 labeled data is demonstrated in Figure 12. The proposed method performs best in ten trials with nearly 97% average accuracy in fault recognition compared with AL-SNN (88.56%), AL-SVM (91.6%), and AL-BPNN (88.9%), indicating that the proposed method can effectively obtain potential information using deep architecture and feature learning as well as detect unknown faults. The BPNN-based method falls into local optimum in the eighth trial, demonstrating the poor stability and convergence of BPNN caused by the lack of unsupervised pre-training.

**Figure 13:**
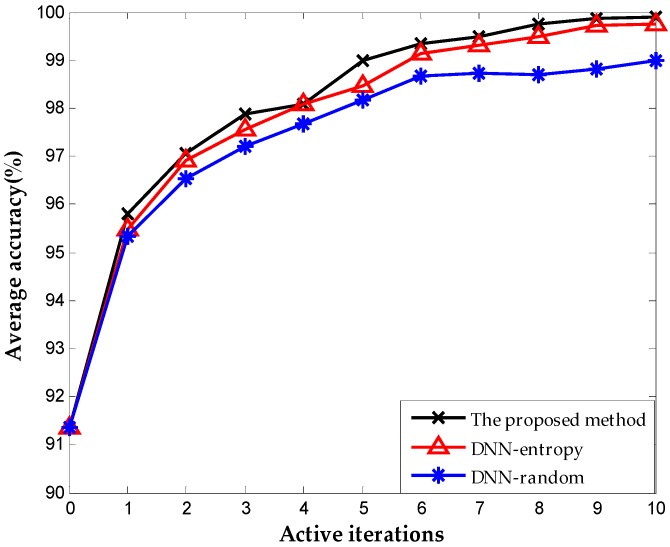
Classification accuracy obtained on TE dataset.

**Figure 14:**
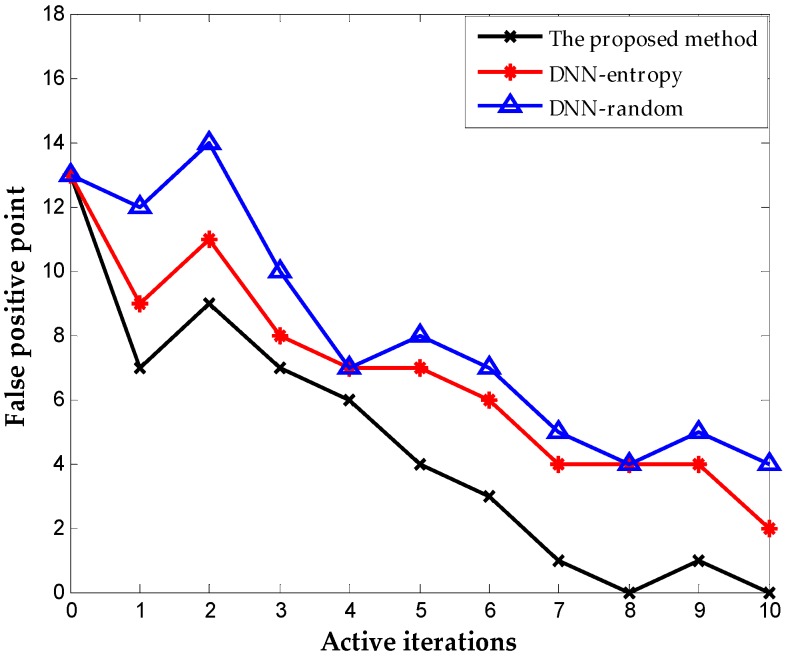
Number of false-positive point obtained on TE dataset.

Figure 13 shows the results with different selection criterion for DNN fine-tuning. The number of samples for fine-tuning is 60 initially. We update the weights of DNN by fine-tuning 20 new samples obtained by different selection criteria. The result presents that the proposed method provides better performance than DNN-entropy and DNN-random, although they are both based on the DNN method. The accuracies of the three methods reach 99.76%, 99.48%, and 98.2% respectively after 10 iterations. The performance improved by approximately 1.5% with the employment of active learning. As indicated in Figure 14, the proposed method performs best in the inhibition of false positive that leads to the running with failure in chemical systems. It is worth illustrating that some fluctuations occurred on the effect of improving false positive using the proposed method (in the second iteration, the false positive rate increase suddenly, which is most probably caused by the randomness of the neural network). The improvement becomes stable with the increase in iterations, and outperforming the other two methods overall.

Table 3 presents the details of the fault diagnosis on the TE dataset after three iterations with the different methods mentioned above. It is indicated that the accuracies in different fault types vary somewhat, whereas the proposed method performs steadily and is superior to other methods. The proposed method also performs better in unknown type of fault, which can be seen in the diagnosis result of type 7. SVM- and SNN-based methods show limitation in the recognition of types 2, 3 and 5. The information extraction capability of the proposed method facing the complex chemical system demonstrated in case studies that possessing multidimensional heterogeneous sensory signal and highly nonlinear relationships between diagnosis inputs and the results is verified. 

### 4.4. Discussion

The main contribution of this study is the construction of DNN with active learning to realize a reliable and effective fault diagnosis with chemical sensor data. The proposed method is a novel idea that utilizes deep learning with big chemical sensor data for both fault feature extraction and intelligent fault diagnosis in chemical processes. An active learning criterion achieves the selection of the most valuable sensor data for inducing the DNN model which is a novel active learning method compared with available active learning criterion for the cost-effective selection of collecting sensor data to be labeled, and improves the model performance maximally. We validate the method on two well-known industrial datasets. The results obtained show that the proposed method provides significant improvements in diagnosis accuracy and false positive compared with the existing methods. 

The proposed method is compared with state-of-the-art methods in two perspectives, namely, in that of the model and criterion of sample selection. For the diagnosis models, we have taken two widely used algorithms which have shallow architectures for comparison, namely, a general ANN-based and SVM-based methods, and the two method that have shallow architecture have been employed in TE dataset that can be found in [10,11,41]. Meanwhile, BPNN, which shares the same architecture and trained by the same parameters as DNN, is also added to the comparisons to further demonstrate the performance of deep learning in the construction of DNN. The results above show that the proposed method obtains significantly better diagnosis accuracies and stability than the methods with shallow architectures and BPNN-based method. For sample selection criterion, we include the methods called random selection and entropy selection criteria in the DNN for comparison. Random selection implies to abandon the active learning that only DNN employed, while entropy selection criterion means the development of entropy criterion with DNN, and the employment of entropy criterion can be found in [29]. The results obtained in two datasets demonstrate that the proposed method is robust and efficient during the iterative labeling process and performs better in terms of diagnosis accuracies and the improvement of false positive. Therefore, deep learning technique is particularly suitable for chemical fault diagnosis and not only in the field of pattern recognition. The combination of the DNN with active learning strengthens the efficiency and feasibility of fault diagnosis in chemical processes. 

**Figure 15:**
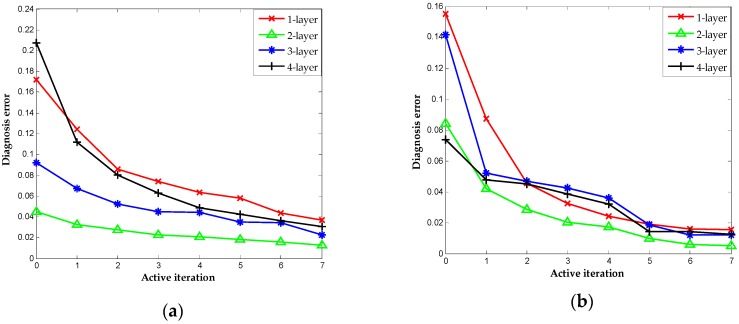
Classification results obtained by AL-DNN with multiple layers: (**a**) UCI (University of California Irvine) dataset; and (**b**) TE dataset.

The number of hidden layer in DNN has a significant effect on performance, owing to the feature learning in hidden layer works is the basis of DNN [9]. We repeat the above experiments with the different configurations of hidden layers to assess the capability of proposed method further. We consider the diagnosis result with the hidden layer settings to 1, 2, 3 and 4 in two datasets. Table 4 shows the details of each configuration. The results correspond to different configurations of the hidden layer, as indicated in Figure 15a,b. It is worth noting that the active learning criteria here are the same and the iterations signify the increase of labeled sensor data for fine-tuning. The configurations with two hidden layers perform best in terms of diagnosis accuracy. In contrast, the scenarios based on one hidden layer have limitations in terms of information extracted that lead to less accurate results. The increase in the number of hidden layer results in poorer performances in these case studies, suggesting the balance between the sensor data and complexity of architectures. 

The criteria of active learning are manifold and have their own advantages in different scenarios, which require the further summarization for different occasions. Furthermore, the construction of DNN is largely dependent on the regulation of parameters, which relies on experience. The architecture selection of DNN is still an open problem that will be investigated further in the future work.

## 5. Conclusions

This study presented a DNN with active learning for chemical fault diagnosis using chemical sensor data. Deep learning technique is employed as a novel method provided for feature representation of original sensor data. The DNN model employs a deep structure with multiple SDAE and works through a hierarchical successive learning process to construct the diagnosis models. A novel active learning criterion for the particularity of chemical process, that is, a combination of BvSB and LFP, is applied in combination with DNN for further fine-tuning that improves the performance of the model in an active manner, which achieves the selection of the most valuable sensor data for inducing the DNN model during the interaction phase. This approach shows several desirable proprieties:

(1) It is able to adaptively mine the feature from the measured sensor signals or data by multiple non-linear transformations and approximate non-linear functions that provide more potential information for various diagnosis issues;
(2) It is an efficient approach for the use of unlabeled sensor data that improves the nature of the diagnosis model in an unsupervised learning;
(3) It relies on a novel active learning criterion compared with available methods to select the most valuable sensor data and improve the DNN significantly, which requires less labeled sensor data during the iterative labeling process.

Compared with the state-of-the art methods on two well-known datasets, the proposed method is able to achieve fault diagnosis with high performance and utilize labeled sensor data effectively. Moreover, this method performs excellently, and is especially superior in diagnosis accuracies and improvement of false positive with less labeled sensor data. Accordingly, the proposed method exhibits significant applicability and potential for fault diagnosis in complex chemical systems that involve multidimensional heterogeneous sensory signal and highly nonlinear relationships between the original senor data and diagnosis results through an effective manner of data utilization. In future research, we also intend to investigate the combination of DNN and parameter optimization technique further.

## Figures and Tables

**Table 1:** Description of fault.

Description	Type
A/C feed ratio, B composition constant	1
B composition, A/C ration constant	2
Reactor cooling water inlet temperature	3
Condenser cooling water inlet temperature	4
A feed loss	5
C header pressure loss-reduced availability	6
Unknown fault	7

**Table 2:** Parameters of DNN.

Parameter	DNN for Case Study 1	DNN for Case Study 2
Learning rate	0.1	0.05
Mini-batch	100	50
Momentum	0.9	0.9
Number of epoch	100	100
Coefficient of sparsity penalty	0.05	0.05
Noise level	0.5	0.1

**Table 3:** Classification results obtained on the TE dataset.

Fault Type	Type 1	Type 2	Type 3	Type 4	Type 5	Type 6	Type 7
The proposed method	99.09%	98.69%	99.18%	94.78%	100%	100%	96.87%
DNN-entropy	100%	99.26%	99.71%	92.75%	99.69%	98.13%	96.24%
DNN-random	97.98%	99.80%	96.34%	95.32%	100%	96.11%	95.98%
AL-BPNN	95.06%	82.60%	89.86%	93.03%	94.24%	97.57%	91.03%
AL-SNN	97.26%	77.56%	75.36%	87.10%	84.37%	94.56%	89.26%
AL-SVM	95.14%	86.94%	90.53%	90.02%	91.57%	95.67%	92.31%

**Table 4:** The parameters of hidden layer.

Configuration	Case Study 1	Case Study 2
The unit number of hidden layer	{100}	{100}
The unit number of hidden layer	{200,100}	{100,50}
The unit number of hidden layer	{200,100,50}	{200,100,50}
The unit number of hidden layer	{300,200,100,50}	{200,100,100,50}
